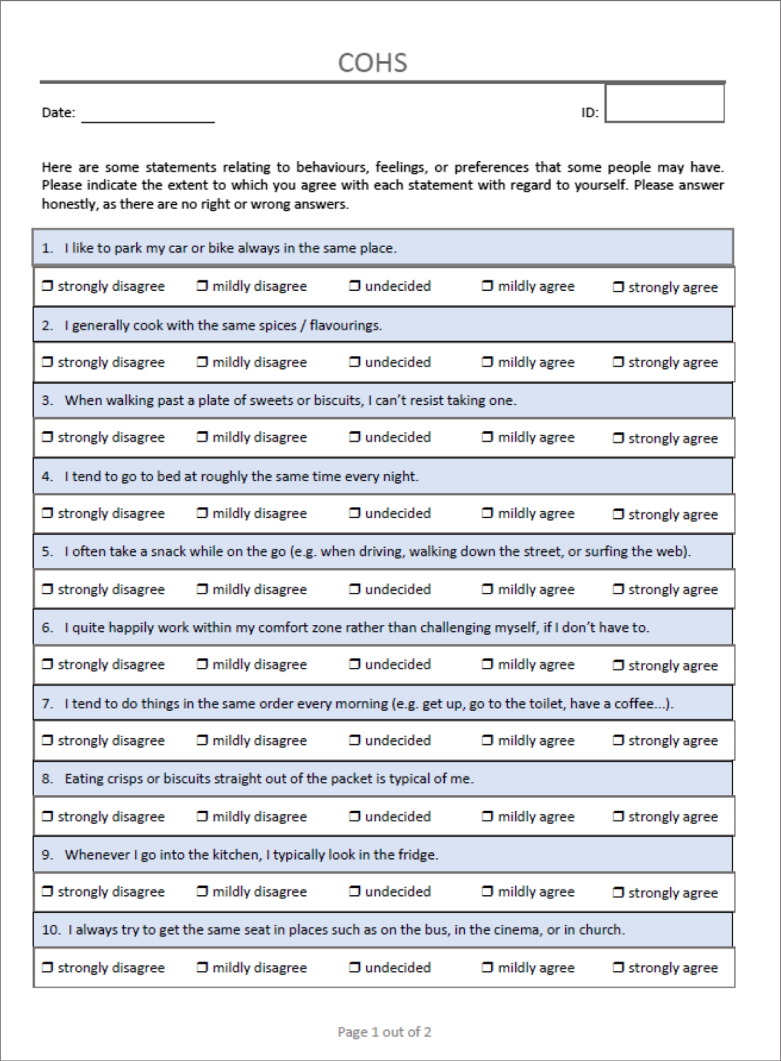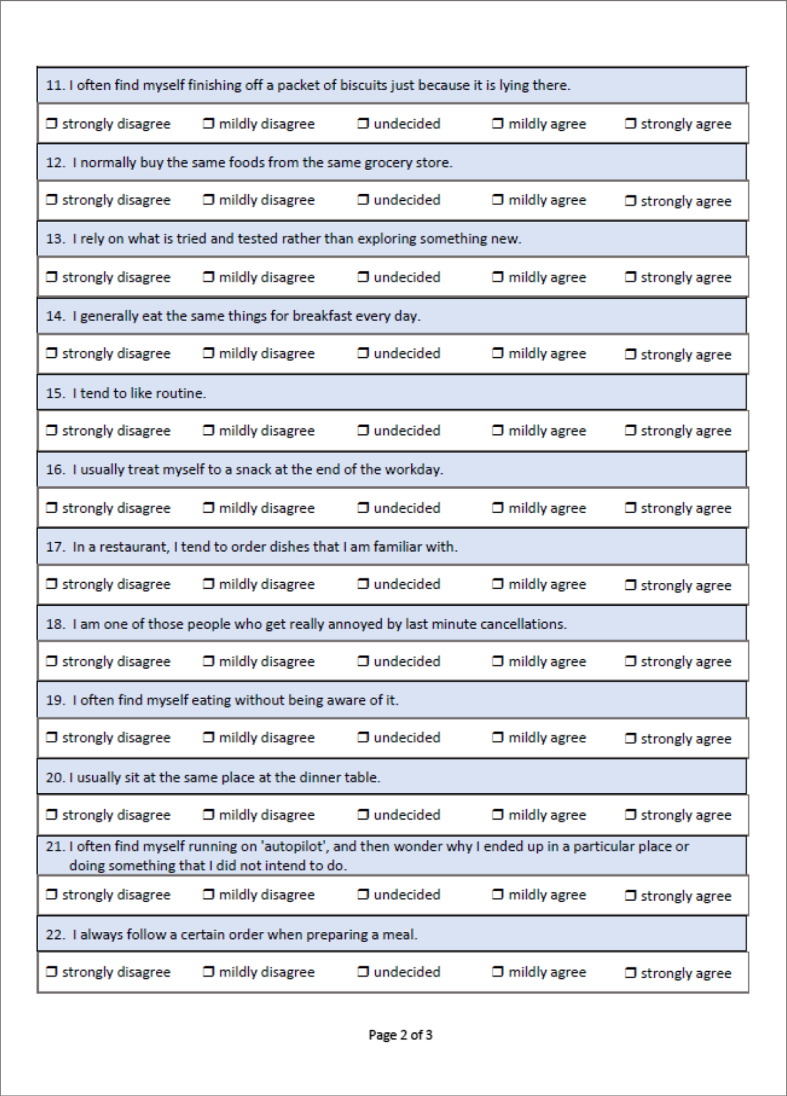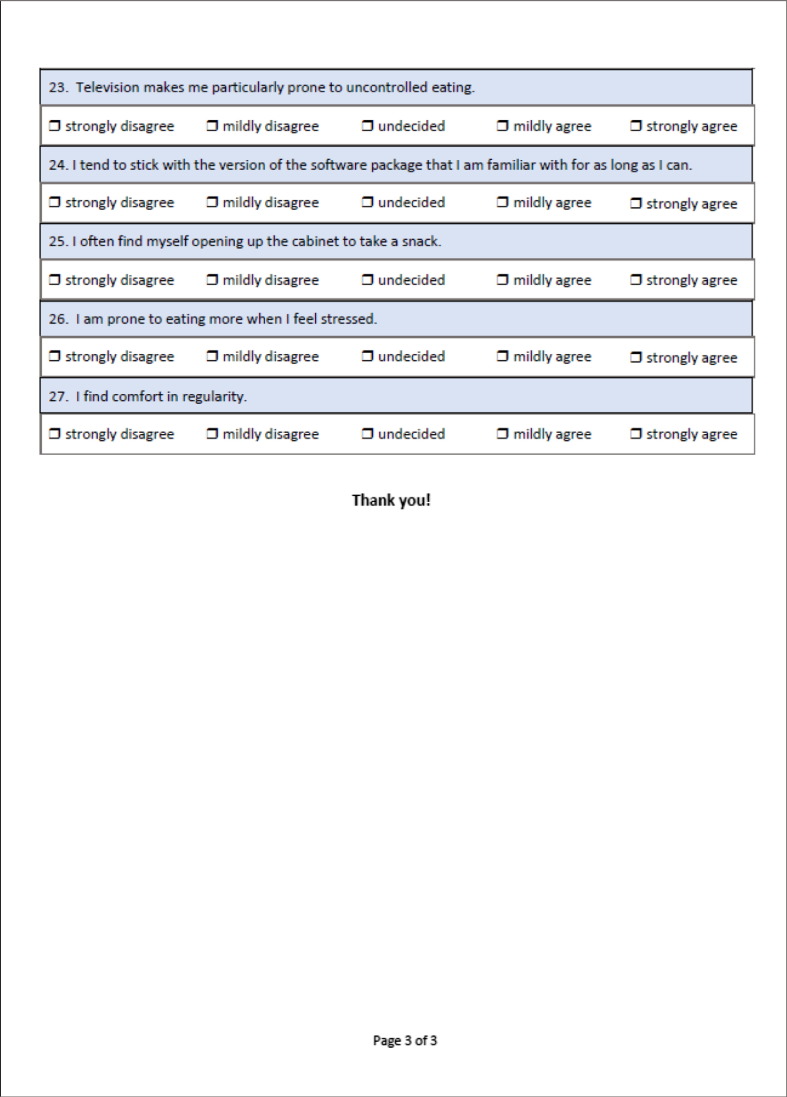# Creature of Habit: A self-report measure of habitual routines and automatic tendencies in everyday life

**DOI:** 10.1016/j.paid.2017.04.024

**Published:** 2017-10-01

**Authors:** Karen D. Ersche, Tsen-Vei Lim, Laetitia H.E. Ward, Trevor W. Robbins, Jan Stochl

**Affiliations:** a Departments of Psychiatry and Psychology, University of Cambridge, Cambridge, UK; b Behavioural and Clinical Neuroscience Institute, University of Cambridge, Cambridge, UK; c Department of Kinanthropology, Charles University, Prague, CZ

**Keywords:** Questionnaire, Daily routines, Automaticity, Eating, Stress, Compulsivity, Sensation-seeking, Stimulant drug exposure

## Abstract

Our daily lives involve high levels of repetition of activities within similar contexts. We buy the same foods from the same grocery store, cook with the same spices, and typically sit at the same place at the dinner table. However, when questioned about these routine activities, most of us barely remember the details of our actions. Habits are automatically triggered behaviours in which we engage without conscious awareness or deliberate control. Although habits help us to operate efficiently, breaking them requires great effort. We have developed a 27-item questionnaire to measure individual differences in habitual responding in everyday life. The Creature of Habit Scale (COHS) incorporates two aspects of the general concept of habits, namely *routine* behaviour and *automatic* responses. Both aspects of habitual behaviour were weakly correlated with underlying anxiety levels, but showed a more substantial difference in relation to goal-oriented motivation. We also observed that experiences of adversity during childhood increased self-reported automaticity, and this effect was further amplified in participants who also reported exposure to stimulant drugs. The COHS is a valid and reliable self-report measure of habits, which may prove useful in a number of contexts where discerning individuals' propensity for habit is beneficial.

## Highlights

• The COHS is a valid and reliable self-report measure of proneness to habits.
• It assesses two aspects of habits: *routine* behaviour and *automatic* responses.
• The routine subscale is predominated by items favouring regularity and familiarity.
• The automaticity subscale is predominated by eating-related items.

## 1 Introduction

Global challenges such as poverty, obesity and climate change require large parts of the general population to change the way we behave in order to make steps towards addressing these problems. Thus far, educational approaches and attempts to appeal to individuals' insight into the pressing need for change have largely failed (Webb & Sheeran, 2006). One reason for the lack of success may be that the targeted behaviours are largely habitual in nature, occurring outside conscious awareness. A better understanding of the mechanisms underlying habitual responses and individual variations in forming and breaking habits is needed in order to develop more effective strategies to address these global challenges (Marteau, Hollands, & Fletcher, 2012).

Habits constitute response patterns that a person repeatedly exhibits in a specific situation (Lally and Gardner, 2013, Wood and Runger, 2016). These responses are learned and become automatically activated when the individual enters the associated environment. Examples could be making breakfast on coming into the kitchen in the morning, or putting the mobile phone onto charge when coming home from work. Such automatic responses are generally triggered by environmental cues, allowing us to perform routine actions highly efficiently whilst focussing our attention on other things. Meanwhile, the original motivation for these habitual actions becomes increasingly irrelevant and, once initiated automatically without intention, habits continue without conscious control. As habits are highly stable, they are difficult to change or break altogether. However, within a different environment (e.g. in a friend's kitchen), the same actions involved in making breakfast may suddenly run less smoothly, requiring conscious attention, and we may likewise run the risk of forgetting to charge the phone at the end of a day off work.

Substantial experimental evidence has shown that habits develop though instrumental learning (Thorndike, 1898). The repetition of reinforced actions, if performed within the same environment, results in contextual stimulus-response associations in memory that trigger the behaviour automatically within that environment (Dickinson, 1985). These stimulus-response associations seem to overshadow the purpose that initially motivated the behaviour, rendering the behaviour insensitive to changes in the value or the contingency of the consequences. When habits are formed, control over the behaviour gradually shifts away from being guided by our intentions to being automatically triggered by cues in the environment. Consequently, once formed, habits are no longer motivated by a goal, and are thus difficult to break with goal-oriented intentions or knowledge of the consequences of habitual actions (Wood & Neal, 2007).

There is significant variation in the degree to which different individuals show a propensity for developing habits. While some people delight in novelty and change in their lives, others go so far as to even describe themselves as ‘creatures of habit’, an expression that reflects their appreciation of routine and regularity in their lives. What underlies these individual differences in habit formation is still largely elusive, but may provide important insight into differences in the strategies needed to change habits in different people. Nevertheless, a number of factors have already been identified that can influence the switch of initially goal-directed actions into habitual responses. These include prolonged practice (Boakes, 1993, Dickinson et al., 1995, Neal et al., 2006), experiences of acute or chronic stress (Dias-Ferreira et al., 2009, Schwabe and Wolf, 2011), or exposure to stimulant drugs (Nelson and Killcross, 2006, Corbit et al., 2014). By contrast, strong executive functions seem to promote goal-directed behaviours (Otto, Raio, Chiang, Phelps, & Daw, 2013), and possibly facilitate the regain of control over behaviours that have become habitual.

A core question meriting consideration revolves around the extent to which ‘creature of habit’ traits might represent a vulnerability marker for the development of clinical conditions in which habitual behaviours have spiralled out of control, such as drug addiction, gambling, obsessive-compulsive disorder, or eating disorders. Indeed, a number of mental health problems involve rigid and inflexible routines, and actions performed in response to particular triggers regardless of negative consequences (American Psychiatric Association, 2013). Clarification around a role for proneness to habits in these conditions may shed light on more successful treatments than those currently available.

Habitual behaviour can be assessed by experimental paradigms that manipulate the value or contingencies of the outcome to identify behaviour patterns that persist irrespective of such manipulations (Ersche et al., 2016, de Wit et al., 2007, Mckim et al., 2016, Gillan et al., 2013). Evidently, self-report measurements of behaviours that largely occur without awareness is not without criticism (Sniehotta & Presseau, 2012). The self-reported habit index (SRHI) is one of the few questionnaires that evaluate individuals' perception of a particular behaviour with respect to frequency, automaticity, efficiency, and self-reference using a 12-item rating scale (Verplanken & Orbell, 2003). The focus of the SRHI lies on a specific recurring behaviour that has been identified by the researcher, not by the scale. This presents a major drawback of the SRHI, as it excludes individuals who, due to a different lifestyle, do not engage in the behaviour in question. To the best of our knowledge, there are currently no tools available to assess more generally how individuals differ in their engagement in habits in daily life.

The aim of the present study, therefore, was to develop a scale that reflects variations in individuals' tendencies towards responding in a habitual manner in everyday life. Variations in proneness to habit may be driven by a need for structure and predictability, which may reassure anxious individuals who worry about uncertainty and the possibility of things going wrong in novel situations (Evans et al., 1997, Connors et al., 2001). We therefore hypothesized that increased habitual tendencies are associated with higher levels of anxiety and obsessive-compulsive traits. Conversely, sensation-seeking traits and goal-striving personalities are likely to run counter to regularity and repetition (Dunn, 2000). We therefore predicted that low levels of habitual behaviours in daily life are associated with high levels of sensation-seeking and goal pursuit. An ancillary aim of the study was to examine whether exposure to stress or stimulant drugs, which have been shown to promote habitual responding in experimental settings, also affect participants' self-reported habitual tendencies.

## 2 Methods

### 2.1 Scale development

For the first step in developing a scale measuring characteristic behaviours and attitudes for ‘creature of habit’ traits, we generated a pool of 59 items based on a thorough review of the literature, and interviews and discussions with experimental and health psychologists. On compiling the questionnaire, we noticed that half of the generated items related to tendencies describing regular behaviours (e.g. I park my car always in the same place), mental attitudes surrounding the minimisation of effort (e.g. I quite happily work within my comfort zone), or the establishment of safety/predictability (e.g. I rely on what is tried and tested), as well as emotional reactions when faced with irregularity (e.g. I hate it when the grocery store re-arranges the aisles). The other half of the items were behaviours occurring in the context of eating, such as describing behaviour motivated by preferences (e.g. I have a preferred sandwich), automatic responses (e.g. I always follow a certain order when preparing a meal), and behaviours characterised by a lack of planning (e.g. I tend to cook more than I eat). Participants were required to indicate for each statement their level of agreement on a 5-point Likert scale, ranging from strongly disagree (1) to strongly agree (5). We extensively piloted the questionnaire within the local community and conducted face-to-face interviews about the meaning of the items. Items that were consistently misunderstood were either reworded or removed. Further piloting showed that administering the entire 59-item questionnaire presented a challenge to participants, so that we subsequently divided it into two parts. Although the categorisation of general habits and food-related habitual responses was initially unintended, it provided a rationale for splitting the COHS into two parts with similar numbers of items (see Appendix A).

### 2.2 Study sample

We used Amazon's Mechanical Turk (MTurk), a crowdsourcing internet marketplace, to collect data from 406 individuals in the online community. Forty-four participants (11%) were excluded due to either incompletion, invalid responses or duplication of data, leaving a total sample of 362 participants (47% male), whose identity remained anonymous to the research team. Participants had to be at least 18 years of age [mean age 39.7 years ± 11.5 standard deviation (SD)] and based in the United States of America. All participants received $2.00 for completion of the study, which included the two parts of the COHS with the items in each part being presented in random order, and a selection of validated questionnaires to assess personality traits of anxiety, compulsivity, sensation-seeking, and goal-pursuit. We also collected background information, including ethnicity, native language, education level, and employment status. Moreover, we asked participants to indicate whether they have ever had any experience with stimulant drugs (either for recreational purposes or as medication) and to complete the Childhood Trauma Questionnaire (CTQ, Bernstein et al., 2003). The characteristics of the full study sample and the subgroups are shown in Table 1. As recommended by Meade and Craig (Meade & Craig, 2012), we also included two attention check items to safeguard against careless participants. The study was approved by the Psychology Research Ethics Committee (Pre.2015.124; PI:KDE).

**Table 1:** Key characteristics of study participants in the full sample and the subgroups. [Education level: ^#^ completed College/High School, *completed a University Degree].

	Full sample	No stimulant exposure	Stimulant exposure	No/mild adversity	Moderate to severe adversity
Size (N)	362	255	107	234	128
Age, mean (years)	39.7 (± 11.5)	39.8 (± 11.8)	39.5 (± 10.8)	39.1 (± 11.8)	40.7 (± 10.9)
Age range (years)	19–72	22–72	19–68	19–72	23–68

Gender (%)					
Male	47	43	56	53	36
Female	53	57	44	47	64

Ethnic background (%)					
White	79	75	89	78	80
Black	6	8	3	7	5
Asian	8	9	3	9	7
Hispanic	4	4	4	4	3
Mixed	3	4	1	2	5

Native English speaker (%)					
Native	98	97	99	97	98
Non-native	2	3	1	3	2

Education level (%)					
No completed education	1	0	2	1	1
Secondary education^#^	44	43	48	39	54
Higher education^⁎^	55	57	50	60	45

Employment status (%)					
Not in paid work	14	14	15	14	16
Studying/training	3	2	3	3	2
Part time	17	20	9	17	16
Full time	66	64	73	66	66

### 2.3 Personality measures

*Anxiety personality traits:* The trait version of the Spielberger State-Trait Anxiety Inventory (STAI, Spielberger, Gorsuch, Lushene, & Jacobs, 1983) assesses variations in trait anxiety of a long-standing nature. It consists of 20 questions surrounding worry, tension, apprehension, and nervousness that are rated on a 4–point scale ranging from almost never (1) to almost always (4). *Obsessive-compulsive Personality Traits:* The Obsessive-Compulsive Inventory–Revised (OCI-R, Foa et al., 2002) is an 18-item questionnaire to assess obsessive-compulsive symptoms in both clinical and non-clinical samples. Participants rate the degree to which they have been bothered or distressed by obsessive-compulsive symptoms in the past month on ranging from not at all (0) to extremely (4). *Sensation-Seeking Personality Traits:* The Sensation-Seeking Scale Form-V (SSS-V, Zuckerman, 1996) is a widely-used psychological instrument for measuring individuals' need for novel and complex sensations along with the willingness to take risks for the sake of having such experiences. It is composed of a series of 40 pairs of dichotomous statements from which one must be selected. *Goal-Oriented Personality Traits:* The Habitual Self-Control Questionnaire (HSCQ, Schroder, Ollis, & Davies, 2013) is a 14-item instrument measuring variations in people's commitment to completing tasks and their self-perceptions around their drive for goal-pursuit. Participants express their level of agreement to each item on a 5-point Likert scale, ranging from disagree strongly (1) to agree strongly (5).

### 2.4 Psychometric analysis of the creature of habit scale (COHS)

We first applied graph theory networks (Harary, 1969) to the correlation matrix to visualise the structure of the item pool. We then used the Mokken Homogeneity Model (MHM, Mokken, 1971), a non-parametric method based on Item Response Theory, to identify redundant items, meaningful subscales and summary scores (Stochl, Jones, & Croudace, 2012). The goal of this method is to cluster the items into scales that meet the three key assumptions of MHM: unidimensionality, monotonicity, and local independence. The summary scores of the items within a subscale should then allow to order people with respect to severity of the measured trait. Unidimensional subscales from the original item pool (meaning that all items measure the same latent trait) were identified using Loevinger's scalability coefficients (Loevinger, 1947) to assess scalability of a single item in relation to the other items of the scale as expressed by H_i,_ and the scalability of the total scale, as expressed by H. To meet the unidimensionality assumption of MHM, none of the H_i_'s should drop below 0.30 (Hemker, Sijtsma, & Molenaar, 1995). Monotonicity was assessed using methods available in R package *mokken* (van der Ark, 2007, van der Ark, 2012). Local independence assumption states that an individual's responses to items are independent of each other and is usually only examined by critical review of item wording. Items violating any assumption of MHM were discarded from the final set. Structure and construct validity of the final set of items in the COHS was subsequently assessed by confirmatory factor analysis.

Reliability of subscales was assessed by McDonald's omega (McDonald, 1999). For reasons of convention, we also computed Cronbach's alpha (Cronbach, 1951) and estimated the graded response model (Samejima, 1969) in order to examine measurement error in detail. Finally, we evaluated convergent and discriminant validity using Pearson's correlations with personality traits that have been linked to variations in habitual tendencies. The aforementioned analyses were conducted using R (R Core Team, 2016) and the packages *qgraph* (Epskamp, Cramer, Waldorp, Schmittmann, & Borsboom, 2012), *mokken* (van der Ark, 2007, van der Ark, 2012) and *ltm* (Rizopoulos, 2006).

### 2.5 Effects of childhood adversity and stimulant drug exposure

Depending on whether participants had been exposed to stimulant drugs, they were allocated into two categorical subgroups (no stimulants, stimulant exposure), since no further information with respect to amount and duration of use was available. We also divided the sample based on the CTQ scores into participants with no or mild type of adversity experiences and those with moderate to severe adversity experiences, following the method described by Bradley et al. (2008). Possible adverse experiences included verbal assault, humiliation, intimidation, domestic violence, or sexual abuse. We used *t*-tests and chi-square to explore differences in demographics between the subgroups. We also assessed moderating and mediating effects of stimulant drug use between childhood adversity and the two COHS scales (automaticity, routine). Gender, ethnicity and education were included as covariates to control for subgroup differences in these variables.

## 3 Results

### 3.1 Data structure and item correlations of the COHS

**Fig. 1:**
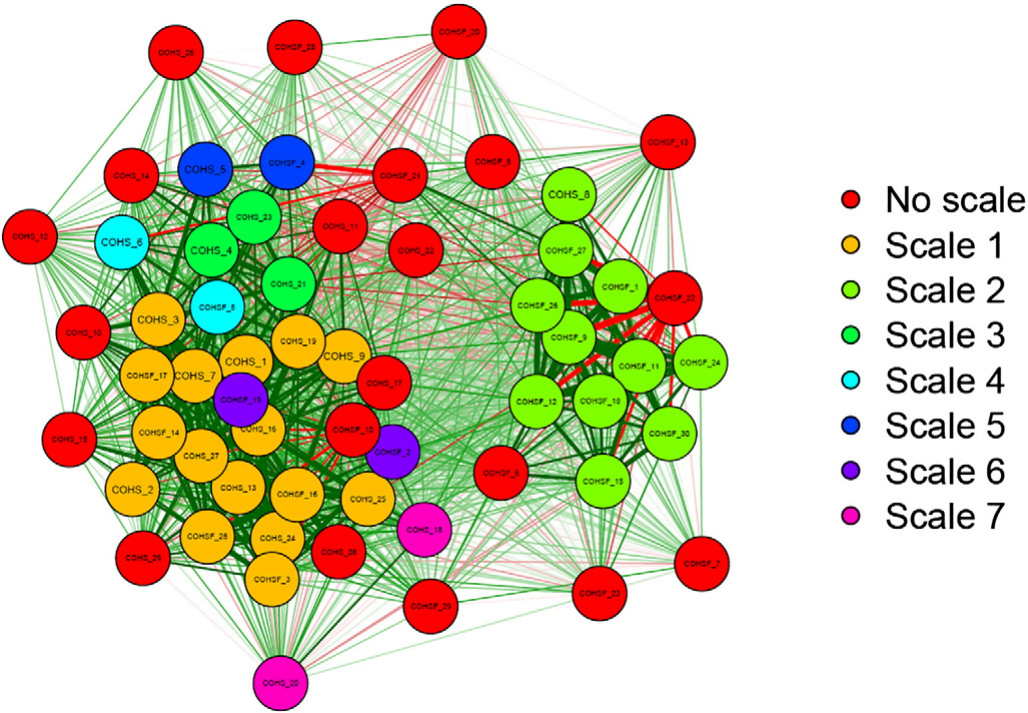
Item correlations and structure of the Creature of Habit Questionnaire. The nodes represent the individual items of the questionnaire. The thickness of lines connecting nodes is proportional to the size of corresponding correlation (correlations of < 0.3 are suppressed for clarity). The layout of nodes is determined by the Fruchterman-Reingold algorithm (Fruchterman & Reingold, 1991); a force-directed layout algorithm that groups highly correlated nodes. The colours represent groupings of items of a subscale, as identified by Mokken Scale Analysis.

Item correlations and structure are shown in Fig. 1 and suggest that items can be roughly divided into two clusters. Mokken scaling indicates that 39 of the 59 items grouped into seven subscales and 20 items failed to either cluster with any subscale or create a subscale on their own. The largest subscale consisted of 16 items, describing behaviours and attitudes that favour order, familiarity and regularity, which we consequently termed *routine*. The second largest subscale included 11 items, creating a relatively separate cluster of items describing automatic behaviour patterns, which we consequently termed *automaticity*. Although the items within the remaining scales 3–7 were strongly correlated, each scale only consisted of just two to three items, which was considered insufficient for a meaningful measurement (Thissen & Wainer, 2001). These items were therefore discarded from the final set, in addition to the 20 items that did not fit into any subscale.

### 3.2 Psychometric properties of the COHS

Table 2 provides additional results from Mokken Scale Analysis on the assessment of homogeneity of subscales. For both scales, homogeneities (H coefficients) were calculated at 0.35 (routine) and 0.41 (automaticity), suggesting medium strength (Sijtsma & Molenaar, 2002). We did not detect any significant violations of monotonicity in either scale, suggesting compliance with the Mokken MHM for all items. Consequently, we ascertained that individuals should be assessed on the basis of the score of each scale separately, rather than by an overall score of both scales (Molenaar, 1982).

**Table 2:** Loevinger's coefficient of homogeneity and scalability (H). Values between 0.30 and 0.40 indicate weak scalability, 0.40–0.50 indicate moderate scalability and 0.50 and above indicate strong scalability. Fifty-nine items were used for the analysis.

COHS - scale 1 (Routine) H = 0.35		H_i_
COHS-16	I tend to like routine.	0.81
COHS-27	I find comfort in regularity.	0.81
COHS-13	I rely on what is tried and tested rather than exploring something new.	0.67
COHS-25	I quite happily work within my comfort zone rather than challenging myself.	0.58
COHS-24	I tend to stick with the version of the software package that I am familiar with for as long as I can.	0.52
COHSF-16	I generally cook with the same spices/flavourings.	0.48
COHSF-28	I normally buy the same foods from the same grocery store.	0.46
COHSF-3	In a restaurant, I tend to order dishes that I am familiar with.	0.45
COHS-7	I tend to do things in the same order every morning (e.g. get up, go to the bathroom, have a coffee…).	0.43
COHS-9	I always try to get the same seat in places such as on the bus, in the cinema, or in church.	0.41
COHSF-14	I usually sit at the same place at the dinner table.	0.4
COHS-1	I like to park my car or bike always in the same place.	0.39
COHSF-17	I always follow a certain order when preparing a meal.	0.38
COHS-20	I am one of those people who get really annoyed by last minute cancellations.	0.36
COHS-3	I tend to go to bed at roughly the same time every night.	0.36
COHS-2	I generally eat the same things for breakfast every day.	0.35


COHS - Scale 2 (Automaticity) H = 0.41		
COHSF-9	I often find myself finishing off a packet of biscuits just because it is lying there.	0.66
COHSF-11	I often find myself opening up the cabinet to take a snack.	0.66
COHSF-12	When walking past a plate of sweets or biscuits, I can't resist taking one.	0.61
COHSF-26	Television makes me particularly prone to uncontrolled eating.	0.57
COHSF-27	I often find myself eating without being aware of it.	0.55
COHSF-1	I am prone to eating more when I feel stressed.	0.52
COHSF-24	Eating crisps or biscuits straight out of the packet is typical of me.	0.5
COHSF-18	Whenever I go into the kitchen, I typically look in the fridge.	0.47
COHSF-15	I usually treat myself to a snack at the end of the workday.	0.45
COHSF-30	I often take a snack while on the go (e.g. when driving, walking down the street, or surfing the web).	0.42
COHS-8	I often find myself running on ‘autopilot’, and then wonder why I ended up in a particular place or doing something that I did not intend to do.	0.41

**Fig. 2:**
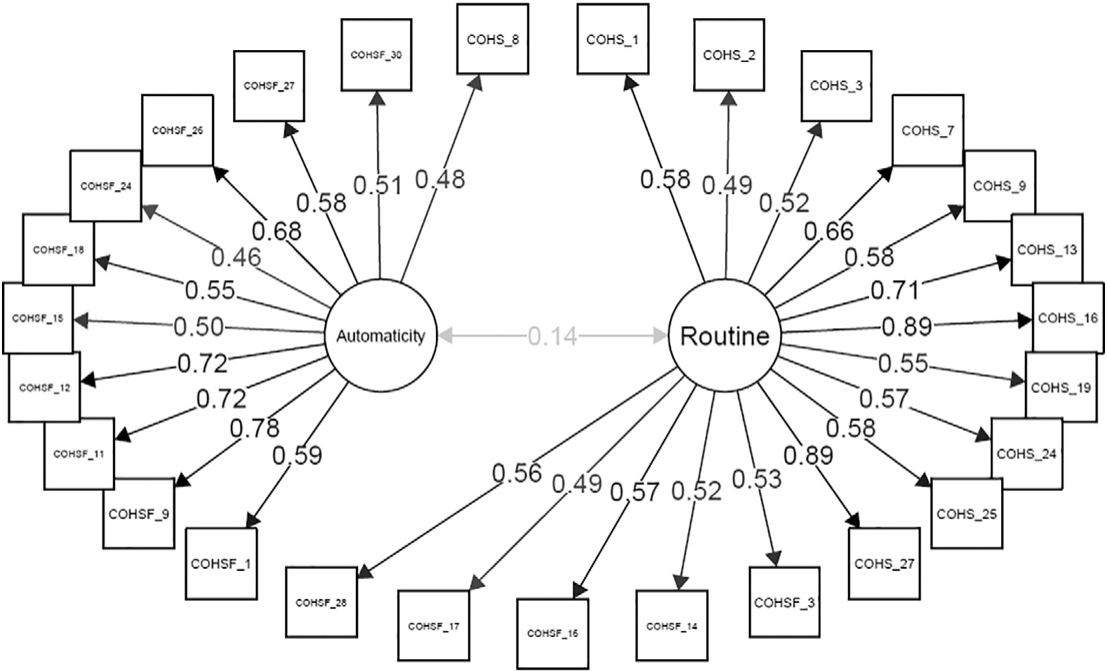
Path diagrams of the COHS, illustrating the strong connections between the individual items and the subscale to which they belong, and the weak relationship between the two subscales.

Both dimensionality of the final item set and construct validity of the items were evaluated using confirmatory factor analysis. Considering the results of the Mokken Scale Analysis and network approach, we hypothesized a 2-factor (correlated) structure for the final item set. The path diagrams of the model are shown in Fig. 2. Model fit was good (Comparative fit index = 0.966, Tucker-Lewis index = 0.963, Root Mean Square Error of Approximation = 0.057 (95% CI 0.051–0.062)). All standardized factor loadings were significant and, with the exception of item COHS_16, higher than 0.5, suggesting good construct validity of items. Factor analysis also confirmed that the two scales, *routine* and *automaticity*, were only weakly correlated domains (*r* = 0.14, *p* < 0.001), which further justified separate scoring of the two scales.

### 3.3 Reliability, measurement error, and validity

**Fig. 3:**
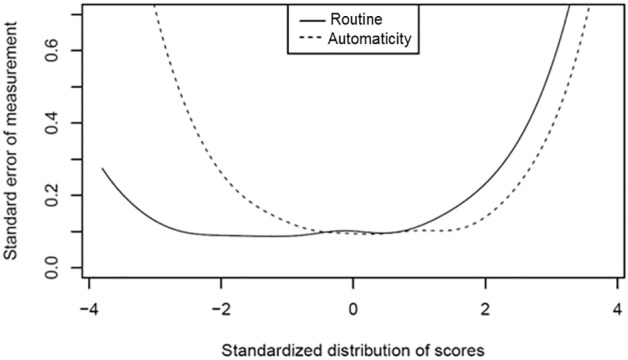
Standard error of measurement as a function of the standardized subscale score distribution.

McDonald‘s omega and Cornbach's alpha for the COHS *routine* (ω = 0.92, α = 0.89) and *automaticity* (ω = 0.91, α = 0.86) suggest satisfactory reliability for both scales. Detailed examination of measurement error can be found in Fig. 3, showing that both scales have reasonably small measurement error across a wide range of score distributions.

As shown in Table 3, the COHS *routine* scale was significantly inversely correlated with sensation-seeking (SSS-V) and weakly correlated with trait levels of anxiety (STAI-T) and compulsivity (OCI-R). COHS *automaticity*, on the other hand, showed a significant inverse relationship with goal-pursuit (HSCQ), in addition to weak correlations with trait-anxiety and compulsivity. We further correlated both subscales with age to examine whether older individuals show a preference for routines (Bergua et al., 2006), but the results were non-significant (both *p* *>* *0.1*).

**Table 3:** Correlations between the two scales of the COHS with selected personality measures (*n* = 362) such as trait-anxiety (STAI-T), goal-pursuit (HSCQ), compulsivity (OCI-R), and sensation-seeking (SSS-V). [Note: ^+^ the correlation coefficient reported here reflects the relationship between the summary scores of each subscale, which differs slightly from the coefficient shown in Fig. 2, which reflects the correlation between two corresponding factors.]

		COHS routine	COHS automaticity	STAI-T total	HSCQ total	OCI-R total	SSS-V total
COHS routine	Pearson correlation	1	0.154^⁎⁎+^	0.246^⁎⁎^	− 0.093	0.265^⁎⁎^	− 0.525^⁎⁎^
	Sig. (2-tailed)		0.003	0.000	0.076	0.000	0.000
COHS automaticity	Pearson correlation	0.154^⁎⁎+^	1	0.370^⁎⁎^	− 0.445^⁎⁎^	0.371^⁎⁎^	− 0.019
	Sig. (2-tailed)	0.003		0.000	0.000	0.000	0.714
STAI-T total score	Pearson correlation	0.246^⁎⁎^	0.370^⁎⁎^	1	− 0.598^⁎⁎^	0.485^⁎⁎^	− 0.050
	Sig. (2-tailed)	0.000	0.000		0.000	0.000	0.346
HSCQ total score	Pearson correlation	− 0.093	− 0.445^⁎⁎^	− 0.598^⁎⁎^	1	− 0.286^⁎⁎^	0.046
	Sig. (2-tailed)	0.076	0.000	0.000		0.000	0.385
OCI-R total score	Pearson correlation	0.265^⁎⁎^	0.371^⁎⁎^	0.485^⁎⁎^	− 0.286^⁎⁎^	1	0.021
	Sig. (2-tailed)	0.000	0.000	0.000	0.000		0.696
SSS-V total score	Pearson correlation	− 0.525^⁎⁎^	− 0.019	− 0.050	0.046	0.021	1
	Sig. (2-tailed)	0.000	0.714	0.346	0.385	0.696	

### 3.4 Effects of childhood adversity and stimulant drug exposure

Participants who reported previous exposure to stimulant drugs were predominantly male (χ^2^ = 5.1,*p* = 0.024) and of white ethnic origin (χ^2^ = 11.2,*p* = 0.047), but were not different from stimulant-naïve participants in terms of education level or employment status. The ANCOVA model, which included gender and ethnicity as covariates, did not reveal any significant subgroup differences in terms of *routine* behaviour (F_1,358_ = 0.1,*p* = 0.921) or *automaticity* (F_1,358_ = 0.8, *p* = 0.365).

For childhood adversity, significantly more women than men reported traumatic experiences during childhood (χ^2^ = 9.7,*p* = 0.002). Participants without adverse childhood experiences were more likely to attend university compared with participants who had such experiences (χ^2^ = 9.0,*p* = 0.011), but they did not differ with respect to ethnicity or employment status.

**Fig. 4:**
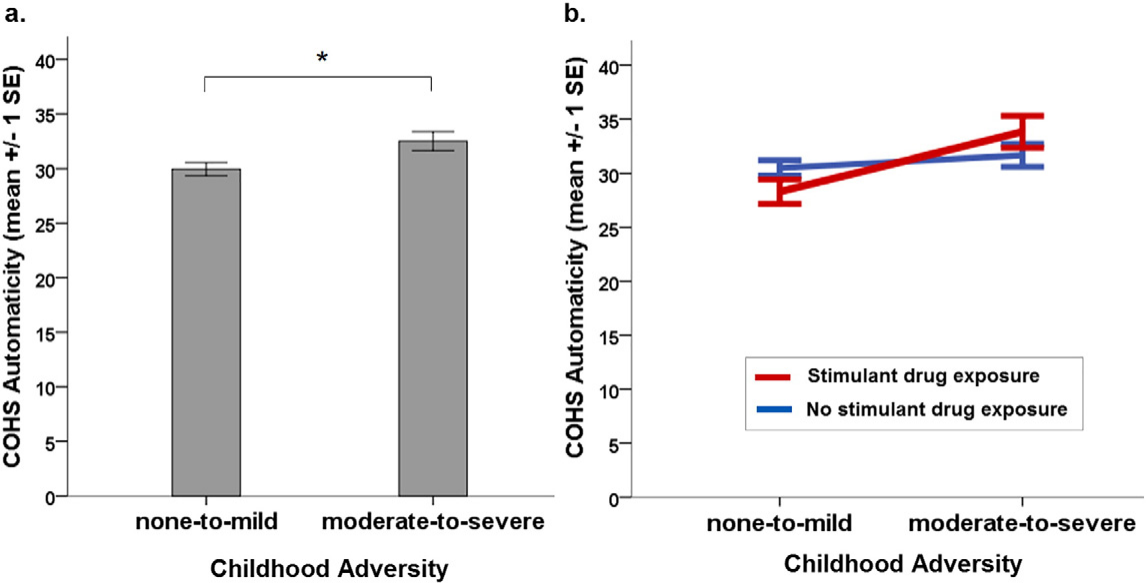
**(a)** Participants reporting experiences of childhood adversity scored significantly higher on the COHS automaticity subscale compared with participants who did not report such experiences. The allocation of participants into the two categories of *none-to-mild* and *moderate-to-severe* levels of experienced adversity was based on the Childhood Trauma Questionnaire (CTQ-SF, Bernstein et al., 2003), following a method described elsewhere (Bradley et al., 2008). **(b)** The effect of childhood adversity on COHS automaticity is further amplified in participants who also reported exposure to stimulant drugs. The subgroups *no stimulant drug exposure* and s*timulant drug exposure* were determined by participants' self-reported lifetime use of stimulant drugs, either for recreational purposes or as medication.

When the two subgroups for childhood adversity were compared, with gender, ethnicity and education included as covariates, participants with childhood adversity showed significantly higher levels of *automaticity* compared with their counterparts without such traumatic experiences (β = 0.126,*p* = 0.019) (Fig. 4). The two subgroups did not, however, differ in terms of *routine* behaviours (β = − 0.018,*p* = 0.746). The comparison of participants with and without stimulant drug exposure, again accounting for covariates, revealed no group differences either for *automaticity* (β = − 0.001,*p* = 0.982) or *routine* (β = − 0.049,*p* = 0.362). However, we did identify a significant interaction effect between stimulant drug exposure and childhood adversity on *automaticity* (β = 0.172, *p* = 0.035) and an interaction effect at trend level for *routine* (β = 0.145,*p* = 0.082) suggesting that stimulant drug exposure further exacerbated the increased levels of automaticity observed in those individuals with childhood adversity. We did not find any mediation effect of stimulant drug exposure on the relationship between childhood adversity and either *automaticity* (total standardized indirect effect = − 0.004,*p* = 0.671) or *routine* (total standardized indirect effect = − 0.007, *p* = 0.426).

## 4 Discussion

We present a novel questionnaire to assess individual variations in habitual tendencies in everyday life. The COHS is theoretically sound and has shown good psychometric properties. As identified by mokken scaling, the COHS differentiates two distinct features of habits: routine behaviours and automatic responses. Questionnaire items that were associated with volition, such as personal preferences or lack of planning, did not survive the analysis, further supporting the notion that habits are not mediated by a goal (Wood & Neal, 2007). The two scales for routines and automaticity both constitute forms of habits: they share with habits their implicit nature, in the sense that within daily life, they may both be initiated without conscious awareness. Habits are behaviours learned by repetition, not by insight (Boakes, 1993) - another critical feature shared by both routines and automaticity. The regular nature of routines involves repetition, and repeated practice is likewise a prerequisite for the transformation of purposeful behaviour into an automatism (Orbell & Verplanken, 2010). Yet, routine and automaticity also distinctly differ from one another, specifically in terms of their function and control over behaviour, as reflected by the weak correlation between the two COHS scales. This differentiation is further supported by the influence that adverse life events and stimulant drug exposure appear to have on the degree to which individuals express automaticity, whilst not affecting routine behaviours.

### 4.1 How routines and automaticity relate to habits

Routines are generally described as familiar action patterns that involve regularity, which are likely to be performed on a daily basis (Gallimore & Lopez, 2002). Routines have a relatively fixed temporal pattern of sequenced actions that are executed voluntarily in order to make daily life more orderly and efficient (Clark, 2000). This functional purpose and their relative independence from the immediate environment are critical features of routines, which may explain why routines are maintained as long as they deliver the desired outcome. Once this is no longer the case, e.g. due to changing circumstances, a conscious decision is typically made to either drop or amend the routine (Clark, 2000, Denham, 2002). Likewise, meaningful routines may also be deliberately combined and turned into rituals in order to increase cohesion between members of a group (Doherty, 1997, Denham, 2003). Although routines fall within the realm of habits, their inherent functionality (either implicit or explicit) and their strong link with a time frame, may explain why routines and habits are *not* synonymous. Habits thus only include those routine behaviours that are performed automatically without serving a specific purpose and are no longer restricted to a fixed temporal pattern.

One possibility is that the degree to which people are inclined to apply routines in their daily lives might be one factor underlying their proneness to form habits. Personality traits associated with seeking or avoiding situations of novelty and excitement may further explain the spread of individual variations in routine practices (Dunn, 2000). Clearly, individuals with high sensation-seeking traits would be less inclined to engage in rituals, which would make them feel bored and stuck in a rut, whilst those more prone to anxiety in novel situations may relish the predictability and comfort of routine.

Just as routine behavioural patterns can reduce effort and cognitive load, so can automatic behaviours, thereby enhancing functional efficiency of daily activities. However, in contrast to routines, automaticity neither has to be sequential in nature nor does it involve any kind of deliberation, cognitive direction or dependency on utility of the outcome (Saling & Phillips, 2007). In fact, automatic actions are initiated by environmental cues without a deliberate intention, and they may even continue without the involvement of conscious control (Bargh, 1994). Habits, by contrast, extend beyond simple automatic reactions, involving complex patterns of behaviours that are performed repeatedly and relatively automatically with very little variation (Clark, 2000). Environmental cues not only trigger the behavioural response but also the mental representation of the habit (Aarts and Dijksterhuis, 2000, Wood and Runger, 2016). This may also explain the inverse relationship between automaticity and goal-striving in the COHS. As such, goal-striving individuals manage their behaviour with consideration for the consequences of their actions in mind, and are less inclined to allow environmental stimuli to take over control.

Given that automatic actions are dependent on the context that triggers them, automatic action patterns necessarily vary enormously with respect to individuals' lifestyles. This poses a particular challenge for self-report measures, which require questionnaire items that capture the specificity of the environment while applying to a large number of people. As the consumption of food is largely automatic (Cohen & Farley, 2008), it is not surprising that many automaticity items are related to eating. Yet, this should not detract from the fact that other non-food related behaviours are equally likely to be automatic but are just more difficult to assess by questionnaire. It is of further note that eating habits also involve routines, as traditionally exemplified by the eating pattern of three meals a day (Jastran, Bisogni, Sobal, Blake, & Devine, 2009). The involvement of the routine component of habit in food-related behaviour is also captured by a number of items in the routine subscale of the COHS.

### 4.2 The benefits and risks of habits

A major hallmark of habits is that they are not mediated by a goal (Dickinson, 1985, Wood and Neal, 2007). Although goal pursuit might have motivated the behaviour initially, once the habit has been acquired, the goal is no longer required to motivate or guide the actions, thereby making habits less effortful and cognitively demanding than goal-orientated behaviours. Habits thereby allow individuals who are affected by cognitive decline or impaired motivation, such as older adults or drug-addicted individuals, to behave highly efficiently despite their deficits (Bergua et al., 2006, de Wit et al., 2014, Ersche et al., 2016), albeit at the expense of flexibility. Furthermore, as habits are decoupled from goal-orientated motivation, habitual behaviour is less susceptible to motivational urges, offering therapeutic opportunities for individuals who particularly struggle with resisting temptations and cravings (Lin, Wood, & Monterosso, 2016). This critical component of habits, namely their insensitivity to reinforcement contingencies or outcome, is, however, not captured by the COHS, but may be explored by studies using the COHS together with experimental paradigms.

Importantly, although habits continue even though these actions are no longer needed, they do not necessarily continue in an uncontrolled manner; for example, they will not be carried out in exactly the same way when there is a change in the environment or temporal configuration of the situation (Lombo & Gimenez-Amaya, 2014). However, if habits lose the specific link to the context and co-occur with compromised inhibitory control, an increased risk arises of habitual patterns being repeatedly practised over and over again, becoming more and more deeply entrenched, which is the case in clinical conditions involving maladaptive habits such as obsessive-compulsive disorder and addiction (White, 1997, Mishkin et al., 1984, Graybiel and Rauch, 2000). Accordingly, both COHS factors were positively correlated with OCI-R scores, a clinical measure of compulsive symptoms (Foa et al., 2002). Although the present sample was not drawn from a clinical population, the positive relationship suggests that both aspects of habits captured in the COHS lend themselves to susceptibility for development of compulsivity. The underlying causes for the development of compulsivity are still elusive, but investigation into them is of critical importance given their apparent relevance to a number of disorders.

### 4.3 Neural substrates of the creature of habit components

The differentiation of routine behaviour and automaticity, as assessed by the COHS, might also be reflected in distinct neural substrates underpinning different components of habits. Hitherto, the basal ganglia have been considered to play a key role in the formation of habits (Ashby et al., 2010, Yin and Knowlton, 2006, Graybiel, 2008). During the initial phases when a behaviour is learned, the associative part of the striatum (caudate nucleus and anterior putamen) and the adjunct limbic structures and medial prefrontal cortex are critically involved (Doyon et al., 2009, Ashby et al., 2010). In as much as these brain systems are critical for both learning and memorising the instrumental contingencies as well as the differential effects of action-outcomes (Liljeholm et al., 2011, Tanaka et al., 2008), goals remain relevant during this phase and might still be involved in the development of routines. However, with prolonged practice, sensorimotor regions of the striatum (putamen) that are primarily connected to sensory and motor cortices take over control (Tricomi, Balleine, & O'Doherty, 2009), resulting in behaviour patterns becoming more automatic and eventually taken over by the cerebellum, possibly contributing to automaticity (Lang and Bastian, 2002, Doyon et al., 2009). Whether the processes for developing routines and automatic response are reflected in variations in connectivity in cortico-striatal and cortico-cerebellal pathways, respectively, are hypotheses to be tested using the COHS in combination with neuroimaging technology.

### 4.4 Creature of habit: Moderator effects

Participants who reported adverse childhood experiences scored significantly higher on the COHS automaticity scale than those who did not report such experiences. This effect was further exacerbated in individuals who also reported using stimulant drugs at least once in their lives. These results are in keeping with prior preclinical work suggesting that the development of automatic responses could be accelerated by exposure to stress and psychostimulant drugs. Our findings are consistent with preclinical work showing that both stress and stimulant drugs induce sensitisation of dopaminergic systems, thereby promoting a more rapid transition to stimulus-response action patterns (Dias-Ferreira et al., 2009, Nelson and Killcross, 2006, Nordquist et al., 2007). From a psychological perspective, a predominance of automatic responses under stress is clearly beneficial. As long as the context is stable, the individual may rely on cerebellar circuits, ensuring that complex actions are performed efficiently even under stressful conditions, (Doyon et al., 2009). In light of the role automaticity plays during stress, it is noteworthy that most of the COHS automaticity items are food-related, which might point to the stress-induced neuroendocrine functions that have also been linked with automatic eating patterns (Kandiah et al., 2008, Tryon et al., 2013). This further supports our finding of higher automaticity scores in people who report having experienced stressful childhoods.

## 5 Conclusions

The 27-item COHS is a measure of habitual tendencies in daily life that demonstrates good validity and reliability (Appendix B). The questionnaire has been designed to measure creature of habit traits, but the findings also point to sub-dimensions of habits related to automatic responses and routine behaviours. This differentiation sheds light on habitual behaviours and may inform interventions to break habits in populations with abnormal maladaptive habits. For example, it is conceivable that individuals with highly expressed routine behaviours would particularly benefit from training alternative routines to replace maladaptive habits. Conversely, for individuals with a particular affinity for automaticity, interventions would be more effective if they were to focus on breaking the maladaptive stimulus-response relationships by linking the triggering stimulus to a more desirable response and practising this new stimulus-response relationship. A shortcoming of the current questionnaire is that it does not assess the critical feature of habits concerning insensitivity to reinforcer devaluation – a feature that would be difficult to assess by self-report. Thus, further benefit could be derived from administering the COHS in combination with other diagnostic tools, specifically instrumental learning tasks. Moreover, cross-validation of the COHS in an independent sample is also warranted.

Generally, the potential usefulness of this questionnaire may be better appraised when the measure has been used in further empirical studies. The use of neuroimaging methods may be of particular benefit in elucidating the different neural pathways involved. Specifically in populations with dysfunctional habit formation, the COHS may help to clarify which aspects of the construct are abnormal, and may then help to determine the most appropriate therapeutic strategy.

## Conflict of interest

All authors declare that they have no competing or potential conflicts of interest in relation to this work.

## Figures and Tables

**Table:** 

COHS-items reflecting regular behaviours, mental attitudes to minimise effort/increase predictability, and emotional responses when prevented from performing these dispositions
1. I like to park my car or bike always in the same place.
2. I generally eat the same things for breakfast every day.
3. I tend to go to bed at roughly the same time every night.
4. I cannot stand leaving things unfinished.
5. I usually exercise at the same time of day.
6. I wake up at roughly the same time every day, even without the help of an alarm clock.
7. I tend to do things in the same order every morning (e.g. get up, go to the toilet, have a coffee…).
8. I often find myself running on ‘autopilot’, and then wonder why I ended up in a particular place or doing something that I did not intend to do.
9. I always try to get the same seat in places such as on the bus, in the cinema, or in church.
10. I have a nightly wind-down routine that helps me to fall asleep more easily.
11. I get annoyed by people who open a new packet of food or bottle of drink (e.g. milk) before the old one is finished.
12. I have a favourite mug for my tea/coffee.
13. I rely on what is tried and tested rather than exploring something new.
14. I generally keep a spare set of things such as batteries that I can fall back on, if needed.
15. Although sometimes tempted to go for special offers, I generally buy household brands that I can trust.
16. I tend to like routine.
17. I hate it when the grocery store rearranges the aisles.
18. If I could, I would probably always pick the same outfit to wear every day.
19. I am one of those people who get really annoyed by last minute cancellations.
20. I haven't bothered to change my hairstyle for several years.
21. I hate being late for anything.
22. I generally put my phone on charge even when it is more than half-full.
23. I regularly tidy up my workspace.
24. I tend to stick with the version of the software package that I am familiar with for as long as I can.
25. I quite happily work within my comfort zone rather than challenging myself, if I don't have to.
26. If I can, I like to give exact change at the checkout.
27. I find comfort in regularity.
28. I always tend to visit the same websites when browsing the internet.
29. I like to revisit places where I have been before.

COHSF items reflecting food-related habits such as preferences, automatic responses, lack of planning and forethought.
1. I am prone to eating more when I feel stressed.
2. I have a preferred sandwich that I always pick.
3. In a restaurant, I tend to order dishes that I am familiar with.
4. I tend to plan meals days in advance.*
5. I usually eat at certain times of the day, even when I am on holiday.
6. I like to finish meals either with something sweet or something savoury.
7. I always lick the lid of a yoghurt.
8. I typically eat the best part of the meal at the end.
9. I often find myself finishing off a packet of biscuits just because it is lying there.
10. I quickly get bored of preparing the same dishes over and over again.*
11. I often find myself opening up the cabinet to take a snack.
12. When walking past a plate of sweets or biscuits, I can't resist taking one.
13. I tend to cook more than I can eat.
14. I usually sit at the same place at the dinner table.
15. I usually treat myself to a snack at the end of the workday.
16. I generally cook with the same spices/flavourings.
17. I always follow a certain order when preparing a meal.
18. Whenever I go into the kitchen, I typically look in the fridge.
19. I like to eat some foods in a certain way (e.g. I eat the pizza crust first or cut up all the meat on the plate in one go).
20. I always add salt before tasting food.
21. When I come home, I often have no idea what to have for dinner.
22. I only eat when I am hungry.*
23. I always need something to drink with a meal.
24. Eating crisps or biscuits straight out of the packet is typical of me.
25. It feels odd not having both a knife and fork for eating meals.
26. Television makes me particularly prone to uncontrolled eating.
27. I often find myself eating without being aware of it.
28. I normally buy the same foods from the same grocery store.
29. Finishing everything on my plate is typical of me.
30. I often take a snack while on the go (e.g. when driving, walking down the street, or surfing the web).

*Contra-indicative items were reversed scored.

**Figure:**